# Coexisting and Second Primary Cancers in Patients with Uveal Melanoma: A 10-Year Nationwide Database Analysis

**DOI:** 10.3390/jcm10204744

**Published:** 2021-10-15

**Authors:** Yong Joon Kim, Myeongjee Lee, Eun Hwa Kim, Inkyung Jung, Christopher Seungkyu Lee

**Affiliations:** 1 Department of Ophthalmology, The Institute of Vision Research, Yonsei University College of Medicine, Seoul 03722, Korea; kyjcolor@yuhs.ac; 2 Biostatistics Collaboration Unit, Department of Biomedical Systems Informatics, Yonsei University College of Medicine, Seoul 03722, Korea; mlee1004@yuhs.ac (M.L.); ehkim0607@yuhs.ac (E.H.K.); 3 Division of Biostatistics, Department of Biomedical Systems Informatics, Yonsei University College of Medicine, Seoul 03722, Korea

**Keywords:** uveal melanoma, coexisting cancer, second primary cancer, standard incidence rate

## Abstract

Uveal melanoma is the most common intraocular tumor in adults. Metastatic disease occurs in about 30% of patients, for which there is currently no effective treatment. More than half of patients are long-term survivors, and it is well established that cancer survivors are prone to developing second primary cancers. In this study, we analyzed 10 years’ worth of data from the nationwide database to determine the rates of coexisting malignancies and second primary cancers associated with uveal melanoma. The mean annual incidence of uveal melanoma was 1.1 per million. Approximately 43% of patients had coexisting cancers. The most common coexisting cancer was lung cancer (10%) followed by liver cancer (6%) and non-Hodgkin lymphoma (6%). In patients whose first cancer in their lifetime was uveal melanoma, the 10-year cumulative incidence of second primary cancers was 22% (95% confidence interval, 9–31%). The age- and sex-adjusted standard incidence rates was 3.61 (95% confidence interval, 2.61–4.86). The most common second primary cancers were lung cancer and hepatocellular carcinoma, followed by prostate, thyroid, pancreatic, and ovarian cancers. Age was the only factor associated with second primary cancer development. Our findings will be helpful in providing counseling for cancer screening in uveal melanoma patients.

## 1. Introduction

Uveal melanoma is the most common intraocular tumor in adults [1,2]. Although several of the signaling pathways involved in uveal melanoma have been discovered, this has not translated into an increase in survival rate [3,4,5,6,7]. While primary uveal melanomas respond well to radiotherapy, liver metastasis occurs in about 30% of patients, and most patients with metastatic disease die within 2 years [8]. About 70% of uveal melanoma patients are the long-term survivors [9].

Many cancer survivors have coexisting cancers and second primary cancers (SPCs), a phenomenon ascribed to both environmental factors, such as smoking and occupation, and genetic factors [10,11,12,13,14]. For example, patients with familial adenomatous polyposis frequently develop colon cancer, osteoma, adrenal carcinoma, and thyroid cancer due to the increased β-catenin activity caused by the *APC* gene mutation [15,16,17]. Women positive for the *BRCA1/2* gene mutation frequently develop hereditary breast and ovarian cancers [18,19]. In this context, identifying cancers that accompany uveal melanoma could help to elucidate the underlying mechanism of uveal melanoma and enable counseling provisions for cancer screening.

Previous studies have reported that between 7.7% and 16.1% of patients with uveal melanoma develop SPCs including skin, kidney, breast, and prostate cancers [20,21,22,23]. Most of these studies were published before 2010, thus the incidence may have been underestimated considering the subsequent development of cancer screening methods. In South Korea, a national cancer screening program was introduced in 1999 and was expanded upon thereafter. This program enables the more accurate analysis of the incidence of coexisting cancers and SPCs. In this study, we analyzed 10 years’ worth of data from this nationwide database to determine the rates of coexisting malignancies and SPCs associated with uveal melanoma.

## 2. Materials and Methods

### 2.1. Data Sources

This was a nationwide, retrospective, cohort study based on the Korean Health Insurance and Review Assessment (HIRA) database, which comprises nationwide health insurance claim data covering approximately 98% of the total population of Korea and including the general characteristics of the beneficiaries as well as all diagnoses, procedures, treatments received in health care services, and inpatient and outpatient prescriptions [24]. In the HIRA database, all beneficiary and health care provider identifications are encrypted according to the Health Insurance Portability and Accountability Act privacy rule to protect personal information, and diagnoses are coded based on the *International Classification of Diseases 10th Revision* (ICD-10). This study was approved by the Institutional Review Board (IRB)/Ethics Committee of Severance Hospital, Yonsei University Health System (IRB No. 4-2019-0618), which also waived the requirement for informed patient consent due to the retrospective study design and use of de-identified data.

### 2.2. Study Population

Uveal melanoma patients are defined by ICD-10 code C69.3 or C69.4 as the principal or first additional diagnosis with codes for expanding benefit coverage (V-code in Korea). Policies to expand benefit coverage commenced in 2005 and have reduced the medical expenses for catastrophic illnesses, such as cancer and cardiocerebrovascular diseases [25]. During the study period (January 2008 to December 2018), 702 patients with uveal melanoma were identified. A 1-year washout period was applied to exclude prevalent uveal melanoma cases (*n* = 123). We further excluded seven patients with a diagnosis of retinoblastoma as the primary cancer. Therefore, 572 uveal melanoma patients were included in the analyses.

### 2.3. Definition of Coexisting Cancer and SPC

The objectives of this study were to identify coexisting cancers accompanying uveal melanoma and to determine the incidence of SPC in patients with uveal melanoma. Coexisting cancer was defined as any malignancy diagnosed prior to or after the diagnosis of uveal melanoma. SPCs were evaluated among patients diagnosed with uveal melanoma as the first cancer in their lifetime. We defined SPC as any cancer that developed at least 6 months after the date of uveal melanoma diagnosis. In these two analyses, skin melanoma (C43), ocular tumors (C69), and secondary cancers in which the involved site was not specified (C77–80) were not considered events of interest. Hematological cancers included Hodgkin lymphoma (C81), non-Hodgkin lymphoma (C82–86 and C97), multiple myeloma (C90), and leukemia (C91–95). Head and neck cancers included palate (C5), mouth (C6), parotid gland (C7), unspecified major salivary gland (C8), tonsil (C9), oropharynx (C10), nasopharynx (C11), unspecified lip, oral cavity, and pharynx (C14), and nasal cavity and middle ear (C30) cancers.

### 2.4. Statistical Analysis

Continuous variables were presented as mean ± standard deviation, and categorical variables are presented as frequencies and their percentages. Age at uveal melanoma diagnosis and the trend of the incidence rate of uveal melanoma during the study period were examined using a linear regression model. In the SPC analysis, only patients diagnosed with uveal melanoma as the primary cancer were included. These uveal melanoma patients were followed up from the date of uveal melanoma diagnosis to the date of SPC diagnosis (event of interest), death, or the end of the study (31 December 2018), whichever came first. The Kaplan–Meier method was used to estimate the cumulative incidence of SPC in uveal melanoma patients. To investigate a potentially increased risk of SPC in uveal melanoma patients compared to the general population, we calculated the standardized incidence ratio (SIR) as the ratio of the numbers of observed to expected cases. The expected number of cancer cases was calculated as the 5-year age-specific standard incidence rates (SIR) for the general population multiplied by the person-years of uveal melanoma patients, using the cancer incidence rates from 2014 reported by the Korea Central Cancer Registry [26]. The 95% confidence intervals (CIs) of the SIR were calculated using the Poisson distribution. We then assessed the associations of age at uveal melanoma diagnosis, sex, and enucleation with the development of SPC using a Cox proportional hazard regression model. Enucleation was considered a time-varying risk factor. We further implemented the Fine and Gray model to consider death as a competing risk [27]. For each risk factor, we implemented a univariate model first and further investigated the association after adjusting for other risk factors. The results are presented as hazard ratios (HRs) with 95% CIs. In all of the analyses, a two-sided *p*-value < 0.05 was taken to indicate statistical significance. SAS Enterprise Guide version 9.4 (SAS Institute Inc., Cary, NC, USA) and R 3.6.1 (R Foundation for Statistical Computing, Vienna, Austria) were used for all statistical analyses.

## 3. Results

### 3.1. Incidence of Uveal Melanoma

**Figure 1:**
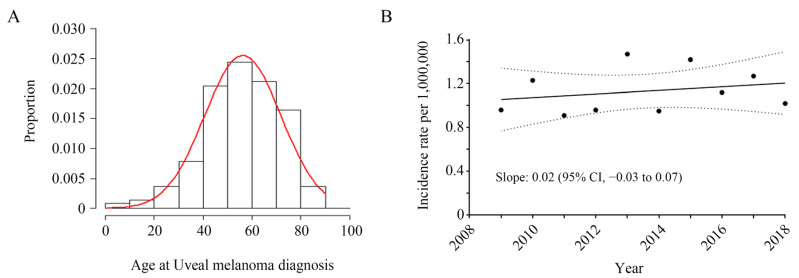
(**A**) Age distribution at diagnosis and (**B**) the annual incidence of uveal melanoma in Korea.

Between January 2008 and December 2018, 702 patients were diagnosed with uveal melanoma and were registered as cancer patients. After the application of a 1-year washout period, 572 patients were included in the analysis. The median age (interquartile range of the 297 (52%) male and 275 (48%) female patients) was 57 (47–67) years of age (Figure 1A). The mean annual incidence rate of disease was 1.1 (range 0.9–1.5) per 1,000,000 people and exhibited an increasing trend over time, but this was not statistically significant (Figure 1B). Among the 572 patients, 501 (88%) and 71 (12%) had choroidal (C69.3) and ciliary body (C69.4) tumors, respectively. During the study period, 352 patients were diagnosed with uveal melanoma as their first cancer.

### 3.2. Coexisting Cancers in Patients with Uveal Melanoma

After excluding melanoma and ocular tumors, 245 (43%) patients were found to have coexisting cancers. Among them, 155 had one coexisting cancer, and 90 had two or more coexisting cancers. The 381 coexisting tumors could be grouped into 51 types of cancers, the most common of which was lung cancer in 58 (10.1%) patients, followed by liver, breast, brain, prostate, and thyroid cancers (Table 1). The coexisting hematological cancers were non-Hodgkin lymphoma in 32 (6%) patients and Hodgkin lymphoma in 4 (1%) patients. There were no patients with leukemia or multiple myeloma. Head and neck cancers were diagnosed in 20 patients.

### 3.3. SPC in Patients with Uveal Melanoma

**Figure 2:**
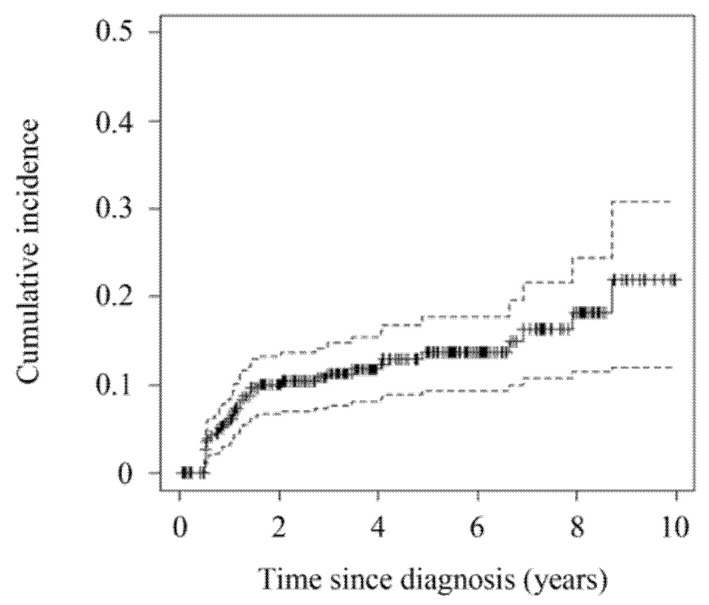
Cumulative incidence of second primary cancers in uveal melanoma patients.

Among the 352 patients for whom uveal melanoma was the first cancer diagnosis, 43 (12%) developed SPCs, of which there were 15 different types (Table 2). The 10-year cumulative incidence of SPCs was 22% (95% CI, 9–31) (Figure 2). The age- and sex-adjusted SIR was 3.61 (95% CI, 2.61–4.86, *p* < 0.001), indicating a significantly higher overall cancer risk in patients with uveal melanoma than in the general population. Data on the observed and expected cases are presented in Table 3. The most common SPCs were lung cancer (9 patients) and hepatocellular carcinoma (9 patients) followed by prostate (5 patients), thyroid (4 patients), pancreatic (3 patients), and ovarian (3 patients) cancer. Most cases of liver, pancreatic, lung, and thyroid cancer developed within the first 2 years after the diagnosis of uveal melanoma. Prostate and ovarian cancers developed after an average of 3.2 (range, 0.8–8.7) and 5.0 (range, 1.4–6.9) years, respectively.

### 3.4. Risk Factors for SPC Development

The associations of age, sex, and enucleation with the development of SPCs were examined. In 352 patients with uveal melanoma as their first cancer, the mean age was 56 ± 14 years, and 182 (52%) were male. Among these patients, 115 (33%) required enucleation during the follow-up period. SPCs developed in 43 (12%) patients, and 44 (13%) patients died. In the Cox models with and without the competing risk model, age was the only factor associated with SPC development (Table 4). Although the hazard ratios were higher for males and those with enucleation, the differences were not statistically significant.

## 4. Discussion

In a nationwide, population-based study, we showed that >40% patients with uveal melanoma had one or more coexisting cancers. This is a higher rate than those in previous studies reporting that ~20% patients with uveal melanoma had coexisting cancers [20,21]. The majority of those studies included patients from the 1960s to 1990s; however, the annual cancer incidence rate and the development of cancer screening methods have increased rapidly over the past 2–3 decades, which may explain the high rate of coexisting cancers identified in the present study. A recent study using the Surveillance, Epidemiology, and End Results database estimated that the long-term cumulative incidence of other primary cancers is >40%, which is similar to the results of our study [22].

The most frequently identified coexisting cancers or SPCs reported by previous studies conducted in white populations of uveal melanoma patients were skin melanoma, non-melanoma skin cancer, and breast, kidney, and prostate cancers [20,21,22,23]. Our analysis showed that uveal melanoma is frequently accompanied by lung, liver, brain, breast, prostate, thyroid, and gastric cancers, all of which are common cancers in Korea. Breast and prostate cancers have also been reported frequently in previous studies in white populations, but the rates of these other cancers differ between previous reports and the present study. These differences in the type of coexisting cancers are thought to be due to racial or environmental factors. Disparities in cancer incidence by race/ethnicity are well established [28]. Melanoma and non-melanoma skin cancers are commonly diagnosed in Caucasian but are rare in Asians [29]. Lung cancer among never smokers is increasing in East Asians, and East Asians harboring *EGFR* gene mutation are far more likely to be diagnosed with lung cancer [28,30]. Long-term exposure to particulate matter may also contribute to the development of lung cancer in Korea. Ambient fine particles are also associated with increased risk of breast, liver, and pancreatic cancers, which are frequently observed co-existing cancers in our study [31]. In terms of gastric cancers, these cancers more frequently and at an earlier age in Asians than in Caucasians, which can ascribed to dietary habits and *Helicobacter pyroli* infection [28,32,33].

Intriguingly, uveal melanoma was found to be frequently accompanied by lymphoma. Few studies have confirmed an association between lymphoma and uveal melanoma, but an association between non-Hodgkin lymphoma and skin melanoma was suggested, with the underlying mechanisms involving exposure to ultraviolet irradiation, commonly shared genetic aberrations, and immune perturbations associated with chemotherapy or comorbidities [34,35,36,37,38,39,40]. A recent large-scale study of >40,000 non-Hodgkin lymphoma patients confirmed that chemotherapy involving fludarabine and T cell activating autoimmune diseases increase the risk of melanoma in non-Hodgkin lymphoma [40]. Our findings suggest that uveal melanoma may also be associated with non-Hodgkin lymphoma. Recent studies have shown that *GNAQ* mutations appear repeatedly in certain types of non-Hodgkin lymphoma, and other studies have suggested that *MYC* alterations are associated with aggressive non-Hodgkin lymphoma or a poor prognosis of uveal melanoma [41,42,43]. In addition, both skin melanoma and uveal melanoma arise from the melanocytes derived from the neural crest, and therefore, immune perturbations may also promote tumorigenesis in uveal melanoma [44]. Further studies are needed to determine whether the incidence of uveal melanoma is actually increased in NHL survivors and to identify the associated risk factors.

This study also analyzed the occurrence of SPCs in addition to coexisting cancers. During the study period, 12% of patients developed SPCs, and the estimated 10-year cumulative incidence was 22%, which was similar to previous studies [22,23]. The calculated SIR in this study was higher than those in previous studies, which was likely due to a number of differences. First, the mean age at onset of uveal melanoma in the Asian populations is the mid-50s, which is significantly younger than the age at onset in white populations. In a previous population-based study, patients diagnosed with uveal melanoma as their first cancer before the age of 50 years had an elevated SPC risk [22]. Second, in Korea, because cancer patients receive a reduction in medical expenses, patients diagnosed with their first cancer undergo more systematic examination, and second cancers may be identified more frequently as a result. Among the risk factors that were evaluated, age was associated with the development of SPCs, with an HR similar to those of previous studies. Male sex and enucleation also increased the HR for SPC, but these associations were not statistically significant.

The main limitations of our study were its retrospective nature and the limitations inherently associated with using a publicly available database. We were unable to include genetic and lifestyle risk factors (e.g., smoking) in the analyses. In addition, as the analysis was conducted based on ICD-10 codes, so the detailed molecular characteristics of the cancers were unknown. Despite these limitations, our data can be regarded as reliable because we defined cancers using both ICD-10 codes and the codes for expanding benefit coverage.

In conclusion, coexisting cancers were observed in more than 40% of patients with uveal melanoma, and associations with lung cancer, liver cancer, and non-Hodgkin lymphoma were confirmed. The incidence of SPCs was 3.61 times higher in uveal melanoma patients than in the reference population. Our findings will be useful in providing counseling for cancer screening in uveal melanoma patients.

## Figures and Tables

**Table 1:** Coexisting cancers associated with uveal melanoma.

ICD-10 Code	Tissue	*n* (%)	ICD-10 Code	Tissue	*n* (%)
C34	Malignant neoplasm of bronchus and lung	58 (10.1%)	C82	Follicular lymphoma	3 (0.5%)
C22	Malignant neoplasm of liver and intrahepatic bile ducts	36 (6.3%)	C20	Malignant neoplasm of rectum	3 (0.5%)
C71	Malignant neoplasm of brain	27 (4.7%)	C70	Malignant neoplasm of meninges	2 (0.3%)
C50	Malignant neoplasm of breast	27 (4.7%)	C40	Malignant neoplasm of bone and articular cartilage of limbs	2 (0.3%)
C61	Malignant neoplasm of prostate	24 (4.2%)	C38	Malignant neoplasm of heart, mediastinum and pleura	2 (0.3%)
C73	Malignant neoplasm of thyroid gland	23 (4.0%)	C37	Malignant neoplasm of thymus	2 (0.3%)
C16	Malignant neoplasm of stomach	17 (3.0%)	C30	Malignant neoplasm of nasal cavity and middle ear	2 (0.3%)
C85	Other and unspecified types of non-Hodgkin lymphoma	16 (2.8%)	C08	Malignant neoplasm of other and unspecified major salivary glands	2 (0.3%)
C44	Other malignant neoplasms of skin	16 (2.8%)	C96	Other and unspecified malignant neoplasms of lymphoid, haematopoietic, and related tissue	1 (0.2%)
C25	Malignant neoplasm of pancreas	10 (1.7%)	C86	Other specified types of T/NK-cell lymphoma	1 (0.2%)
C83	Non-follicular lymphoma	9 (1.6%)	C75	Malignant neoplasm of other endocrine glands and related structures	1 (0.2%)
C72	Malignant neoplasm of spinal cord, cranial nerves and other parts of central nervous system	8 (1.4%)	C62	Malignant neoplasm of testis	1 (0.2%)
C18	Malignant neoplasm of colon	8 (1.4%)	C57	Malignant neoplasm of other and unspecified female genital organs	1 (0.2%)
C76	Malignant neoplasm of other and ill-defined sites	7 (1.2%)	C54	Malignant neoplasm of corpus uteri	1 (0.2%)
C49	Malignant neoplasm of other connective and soft tissue	7 (1.2%)	C53	Malignant neoplasm of cervix uteri	1 (0.2%)
C41	Malignant neoplasm of bone and articular cartilage of other and unspecified sites	6 (1.0%)	C14	Malignant neoplasm of other and ill-defined sites in the lip, oral cavity, and pharynx	1 (0.2%)
C31	Malignant neoplasm of accessory sinuses	6 (1.0%)	C32	Malignant neoplasm of larynx	1 (0.2%)
C67	Malignant neoplasm of bladder	5 (0.9%)	C24	Malignant neoplasm of other and unspecified parts of biliary tract	1 (0.2%)
C64	Malignant neoplasm of kidney, except renal pelvis	5 (0.9%)	C17	Malignant neoplasm of small intestine	1 (0.2%)
C56	Malignant neoplasm of ovary	5 (0.9%)	C15	Malignant neoplasm of oesophagus	1 (0.2%)
C11	Malignant neoplasm of nasopharynx	5 (0.9%)	C33	Malignant neoplasm of trachea	1 (0.2%)
C07	Malignant neoplasm of parotid gland	5 (0.9%)	C10	Malignant neoplasm of oropharynx	1 (0.2%)
C88	Malignant immunoproliferative diseases	4 (0.7%)	C09	Malignant neoplasm of tonsil	1 (0.2%)
C81	Hodgkin lymphoma	4 (0.7%)	C06	Malignant neoplasm of other and unspecified parts of mouth	1 (0.2%)
C74	Malignant neoplasm of adrenal gland	4 (0.7%)	C05	Malignant neoplasm of palate	1 (0.2%)
C19	Malignant neoplasm of rectosigmoid junction	4 (0.7%)			

**Table 2:** Second primary cancers associated with uveal melanoma.

ICD-10 Code	Tissue	*n* (%)
C34	Malignant neoplasm of bronchus and lung	9 (2.6%)
C22	Malignant neoplasm of liver and intrahepatic bile ducts	9 (2.6%)
C61	Malignant neoplasm of prostate	5 (1.4%)
C73	Malignant neoplasm of thyroid gland	4 (1.1%)
C25	Malignant neoplasm of pancreas	3 (0.9%)
C56	Malignant neoplasm of ovary	3 (0.9%)
C71	Malignant neoplasm of brain	2 (0.6%)
C11	Malignant neoplasm of nasopharynx	1 (0.3%)
C16	Malignant neoplasm of stomach	1 (0.3%)
C18	Malignant neoplasm of colon	1 (0.3%)
C41	Malignant neoplasm of bone and articular cartilage of other and unspecified sites	1 (0.3%)
C49	Malignant neoplasm of other connective and soft tissue	1 (0.3%)
C50	Malignant neoplasm of breast	1 (0.3%)
C76	Malignant neoplasm of other and ill-defined sites	1 (0.3%)
C85	Other and unspecified types of non-Hodgkin lymphoma	1 (0.3%)

**Table 3:** Estimation of second primary cancer risk in uveal melanoma patients.

Age Group (Year)	2014 Nationwide Statistics from The Korea Central Cancer Registry			Incidence of Second Primary Cancers in Uveal Melanoma Patients		
	Population of the Middle of the Year	All Cancers (C00-C96)	Incidence Rate (Per 100,000 Person)	Person-Years	Number of Patients with SPC	Expected Number of Patients Developing SPC
0–4	2,297,243.5	460	20	0	0	0
5–9	2,308,229	278	12	0	0	0
10–14	2,731,443	353	12.9	0	0	0
15–19	3,364,378.5	673	20	8.353	0	0.002
20–24	3,433,785.5	1224	35.6	17.268	0	0.006
25–29	3,151,400.5	2409	76.4	25.131	0	0.019
30–34	3,971,975.5	5363	135	61.949	0	0.084
35–39	3,875,142.5	7993	206.3	59.003	0	0.122
40–44	4,509,393.5	13,396	297.1	78.648	5	0.234
45–49	4,301,489.5	16,937	393.7	140.290	5	0.552
50–54	4,322,181.5	23,309	539.3	174.817	1	0.943
55–59	3,678,401.5	25,826	702.1	191.617	7	1.345
60–64	2,521,163	23,716	940.7	151.351	5	1.424
65–69	2,008,782	24,625	1225.90	151.020	4	1.851
70–74	1,785,086.5	27,961	1566.40	144.197	4	2.259
75–79	1,296,333.5	23,273	1795.30	109.865	7	1.972
80–84	729,676	14,052	1925.80	33.139	4	0.638
>85	477,053	8283	1736.30	26.982	1	0.468
Total	50,763,158.0	220131	433.6	1373.629	43	11.919
				SIR for second primary cancers in uveal melanoma: 3.61 (95% CI, 2.61–4.86)		

SPC, second primary cancer; SIR, standardized incidence ratio.

**Table 4:** Factors associated with the development of second primary cancers.

	Cox Model				Cox Model with Competing Risk			
	Univariate		Multivariate		Univariate		Multivariate	
Risk Factors	HR (95% CI)	*p*-Value	HR (95% CI)	*p*-Value	HR (95% CI)	*p*-Value	HR (95% CI)	*p*-Value
Age	1.034 (1.011, 1.057)	0.003	1.036 (1.013, 1.060)	0.002	1.032 (1.010, 1.053)	0.003	1.034 (1.011, 1.057)	0.003
Male	1.492 (0.813, 2.738)	1.196	1.640 (0.890, 3.021)	0.112	1.426 (0.782, 2.599)	0.247	1.545 (0.832, 2.869)	0.168
Enucleation	1.456 (0.776, 2.732)	0.242	1.475 (0.787, 2.767)	0.225	1.433 (0.784, 2.618)	0.243	1.493 (0.824, 2.702)	0.186

End of study: 267 (75%); Development of secondary primary cancers (SPCs): 43 (12%); Death: 44 (12%); HR, Hazard ratio.

## Data Availability

The data presented in this study are available upon request from the corresponding author.
